# Effect of multiple microsporidian infections and temperature stress on the heat shock protein 70 (hsp70) response of the amphipod *Gammarus pulex*

**DOI:** 10.1186/1756-3305-7-170

**Published:** 2014-04-07

**Authors:** Daniel S Grabner, Gerhard Schertzinger, Bernd Sures

**Affiliations:** 1 Aquatic Ecology and Centre for Water and Environmental Research, University of Duisburg-Essen, Universitaetsstr. 5, Essen 45141, Germany

**Keywords:** Microsporidia, Amphipoda, *Gammarus pulex*, Stress response, Temperature stress, Hsp70

## Abstract

**Background:**

Increasing temperatures can be a significant stressor for aquatic organisms. Amphipods are one of the most abundant and functionally important groups of freshwater macroinvertebrates. Therefore, we conducted a laboratory experiment with *Gammarus pulex*, naturally infected with microsporidians.

**Methods:**

In each group, 42 gammarids were exposed to 15°C and 25°C for 24 h. Sex of gammarids was determined and microsporidian infections were detected by specific PCR. To quantify stress levels of the amphipods, the 70 kDa heat shock proteins (hsp70) were analyzed by western blot.

**Results:**

More males than females were detected in the randomized population sample (ratio of females/males: 0.87). No mortality occurred at 15°C, while 42.9% of gammarids died at 25°C. Sequences of three microsporidians (M1, M2, M3) were detected in this *G. pulex* population (99.7%-100% sequence identity to *Microsporidium* spp. from GenBank). Previous studies showed that M3 is vertically transmitted, while M1 and M2 are presumably horizontally transmitted. Prevalences, according to PCR, were 27.0%, 37.8% and 64.9% for *Microsporidium* sp. M1, M2 and M3, respectively. Cumulative prevalence was 82.4%. Multiple infections with all three microsporidians in single gammarids were detected with a prevalence of 8.1%, and bi-infections ranged between 12.2% and 25.7%. In dead gammarids, comparatively low prevalences were noted for M1 (males and females: 11.1%) and M2 (females: 11.1%; males 0%), while prevalence of M3 was higher (females: 66.7%; males: 88.9%). No significant effect of host sex on microsporidian infection was found.

Significant effects of temperature and bi-infection with *Microsporidium* spp. M2 + M3 on hsp70 response were detected by analysis of the whole sample (15°C and 25°C group) and of M2 + M3 bi-infection and gammarid weight when analyzing the 25°C group separately. None of the parameters had a significant effect on hsp70 levels in the 15°C group.

**Conclusion:**

This study shows that some microsporidian infections in amphipods can cause an increase in stress protein level, in addition to other stressors. Although more harmful effects of combined stressors can be expected, experimental evidence suggests that such an increase might possibly have a protective effect for the host against acute temperature stress.

## Background

Microsporidia are a group of highly reduced parasitic fungi found in vertebrates and invertebrates [1-3]. Various microsporidian species commonly infect invertebrates, and can severely impair host fitness, thereby shaping host population size and dynamics [4-8]. Numerous microsporidian species infect amphipods and can compromise their populations either by high virulence causing death of their hosts (e.g. genus *Pleistophora* in gammarids, [9]), or cause a shift in host sex ratio through feminization of the population, either by male killing, or by turning males into functional females [5,10-14]. On the other hand, vertically transmitted microsporidians often do not seem to affect the host populations when measuring mortality [5], but might have effects on behavior [9] or juvenile growth [15,16]. Additionally, microsporidian parasites can influence the response of their amphipod hosts to density dependent factors like food limitation and crowding [15] or environmental contamination with heavy metals [17,18]. If parasitized populations have to face additional stressors, the impact on parasitized individuals might be different compared to uninfected gammarids. Therefore, prediction of stress related population changes is likely to be dependent on infection levels.

Temperature changes, especially increasing temperatures can cause gradual changes in macroinvertebrate communities [19]. On an individual basis, a first response to a temperature rise is an increase in heat shock protein synthesis [20]. Heat shock proteins (hsps) allow organisms to expand their tolerance to a wider range of environmental stressors, for example by acting as molecular chaperones, which bind to denatured and unfolded proteins, prevent aggregation of non-native proteins and repair damaged proteins [21,22]. Under stressful conditions such as high temperature an overexpression of hsps occurs, by which the stress tolerance can be enhanced [23]. To the best of our knowledge, only a limited number of studies exist on the expression of hsps following thermal stress in amphipods [20]. Accordingly, combined effects of temperature change and microsporidian parasite infection on the stress level of amphipods were not studied until now although recent studies suggest profound effects of parasites on hsp-levels in amphipods [24,25]. Thus, it was the aim of the present study to assess effects of microsporidian infection in combination with temperature as an additional stressor in a laboratory experiment with individuals from a population of *Gammarus pulex* from the river Ruhr, Germany, naturally infected with microsporidians. *G. pulex* is a common and widely distributed freshwater amphipod, common in European streams and lakes where it is an important shredder of organic material [26,27]. To date, only limited information is available on microsporidian infections of this species [14,28], and no data exists for microsporidian infections in amphipods from the River Ruhr, North Rhine-Westphalia.

We tested the hypothesis that microsporidian parasites modulate the effect of stressors on their host and addressed the question, if infected individuals are more prone to be affected by extreme environmental conditions. Parasites were detected and characterized by PCR and stress levels were quantified by the response of the 70 kDa heat shock proteins (hsp70).

## Methods

### Temperature experiment

Amphipods were collected from the River Ruhr in July 2013. At this time the river water temperature was 14°C. The species was identified morphologically as *Gammarus pulex* according to the key of Eggers and Martens [29]. Immediately after transfer to the laboratory, single animals were placed into wells of 6-well-plates, each with 10 ml of natural stream water from the collection site and were kept at 15°C with 12 h/12 h light dark cycles for 24 h. Subsequently, the plates were separated into two groups, each comprising 42 individuals. One group was kept at 15°C and one transferred to 25°C for an additional 24 h to induce a stress response to be able to analyze the effect of microsporidian parasites under these conditions. At the end of the experiment, dead gammarids were counted and all animals were frozen in liquid nitrogen and stored at −80°C for molecular and biochemical analyses. The experiment was conducted in compliance with national and institutional guidelines for the care and use of animals.

### Molecular identification of microsporidians

After thawing, sex of gammarids was determined according to male genital papillae or female oostegites. Animals were then weighed and homogenized with micropestles in 0.1 M sodium phosphate buffer with 0.1 M KCl. During processing of the gammarids, care was taken to avoid cross contamination between individuals. Therefore, equipment was rinsed with a 2% sodium hypochlorite solution to remove DNA-contamination. A 30 μl aliquot of the homogenate was used for DNA-extraction, while the remainder was centrifuged at 14.000 × g for 15 min at 4°C. The supernatant was used for quantification of total protein concentration and analysis of 70 kDa heat shock protein (hsp70) (see below).

For molecular detection of microsporidian infections in gammarids, the primers V1 5′-CAC CAG GTT GAT TCT GCC TGA C-3′ [30] and Micro_rev 5′-GAG TCA AAT TAA GCC GCA CAA TCC AC-3′ [31], amplifying a part of the small subunit ribosomal RNA gene (ss rDNA) of microsporidians, were used. Samples were prepared for PCR amplification by heating 2 μl sample in 20 μl dilution buffer and 0.5 μl DNA release additive of the Phire Animal Tissue Direct PCR Kit (Thermo Scientific) for 2 min at 98°C. The mix was centrifuged at 5000×g for 1 min and the supernatant was used for PCR.

To identify species of Microsporidia infecting the gammarids, PCR bands of 10 randomly selected samples were gel purified with a JETQUICK PCR Product Purification Spin Kit (Genomed) according to manufacturer’s instructions, sequenced directly or cloned with the Zero Blunt TOPO PCR Cloning Kit for Sequencing (Invitrogen). Plasmids were purified with peqGold plasmid miniprep kit (peqlab) and 2 clones of each isolate were sent for sequencing (GATC). Additionally, to obtain a longer section of the rDNA (ss rDNA, the internal transcribed spacer and a part of the large subunit rDNA gene (ls rDNA)), the primers V1 and ls580 r 5′-GGT CCG TGT TTC AAG ACG G-3′ [32] were used. All sequences were tested for matches in the GenBank by blast-search (http://blast.ncbi.nlm.nih.gov/Blast.cgi).

According to the sequences obtained, specific primers for each of the three microsporidians species detected were designed (see Table 1) to test the remaining positive samples without the need for sequencing. All PCR reactions contained 10 μl of 2× Phire PCR Buffer, 0.5 μM of each primer, 0.4 μl Phire Hot Start II DNA Polymerase and 1 μl DNA. Water was added to 20 μl. PCR conditions were 98°C for 5 min, 35 cycles of 98°C for 15 s, annealing at 58°C for 15 s, elongation at 72°C for 30s (V1/ls580r) or 20s (all other primers) and a final elongation at 72°C for 2 min. Annealing temperatures for the diagnostic primers are shown in Table 1. PCR bands were analyzed by conventional agarose gel electrophoresis. Specificity of diagnostic primers was assessed by sequencing of three randomly selected PCR products of each primer pair and comparison to the sequences obtained with the primers V1/Micro_rev and ls580r.

**Table 1:** **Specific primers designed in the present study to detect the three microsporidians found in *G. pulex***

**Primer**	**Sequence (5′ - 3′)**	**Annealing Temperature**
Mspec1 F	CAT CAA CTA ACT TTG GGA AAC TAA G	57°C
Mspec1 R	TGG CCT CCC ACA CAT TCC GAG TG	
Mspec2 F	GGC GAT CTA ACC TCG GCA TCG GAT AAC C	64°C
Mspec2 R	TGG CTT CCC ACC CAT TCC GAG C	
Mspec 3 F	CAG TAA TGT TGC GAT GAT TTG GTC	55°C
Mspec3 R	CAG TAA ATA CTC CAC AGT ATC TTA C	

### Analysis of heat shock proteins

Levels of hsp70 were analyzed by the method described in Frank *et al*. [25]. Briefly, total protein was measured in the supernatant with a Pierce BCA Protein Assay Kit (Thermo Scientific). The response of the 70 kDa heat shock proteins (hsp70) was assessed by discontinuous SDS-Page and Western Blot using monoclonal anti hsp70 antibodies (mouse anti hsp70, antibodies online) and a horseradish peroxidase labeled second antibody (goat-anti mouse, DAKO). Gammarids that died during the experiment were excluded from this analysis. For detection of hsp70, 20 μg of total protein were loaded on the gel for each sample. To allow inter-gel comparability, a reference sample (fish liver homogenate) was run on all gels. The hsp70-bands were visualized by 4-chloro-1-naphthol-staining, were scanned and quantified densitometrically with ImageJ [33]. All sample values were divided by the value of the standard of the respective gel and were expressed as relative hsp70 values.

### Statistics

A possible effect of microsporidian infection (M1, M2, M3 and interaction of all three) on host sex was analyzed by a general linear model (GLM) with the binomial model family. Additionally, the effect of the factors “temperature”, “host sex”, “infection with microsporidian 1”, “infection with microsporidian 2”, “infection with microsporidian 3” and interaction of all three, as well as “host weight” on the hsp70 level were analyzed by a linear model. Omission of the parameter “host sex” did not reduce the quality of the model, and was removed during model optimization. The analyses were conducted with R v.3.0.1 [34] and graphs were created with GraphPad Prism v5.0.

## Results

### Gammarids

The ratio of female to male *Gammarus pulex* in the whole sample (15° and 25°C groups) was 0.87, but differed between the two temperature groups with a ratio of 0.91 in the 15°C group and 0.71 in the 25°C group. Sex ratio of both infected and uninfected individuals was 0.87, for the whole population sample (Table 2). No intersex phenotypes (females showing male characteristics) were detected during morphological examination. No mortality occurred among the gammarids of the 15°C group while 42.9% (9 males and 9 females) of the animals died after 24 h exposure to 25°C.

**Table 2:** **Number and ratio of female and male *G. pulex *** **in the different treatments and among infected and uninfected individuals**

	**15°C**	**25°C**	**Dead individuals 25°C**	**Uninfected individuals**	**Infected individuals**	**Total**
Female	20	10	9	7	32	39
Male	22	14	9	8	37	45
Ratio female/male	0.91	0.71	1	0.87	0.87	0.87

### Microsporidian infection

Molecular analyses revealed the presence of three different microsporidian ss rDNA sequence isolates of 1701 bp (M1 [GenBank: KF894401]), 1702 bp (M2 [GenBank: KF894402]) and 763 bp (M3 [GenBank: KF894403]). Amplification of microsporidian M3 with the V1-ss580r primers did not yield a PCR product of sufficient quality, therefore, only the shorter V1/Micro_rev product was sequenced for this species. Blast search and sequence comparison showed 99.7% identity (3 nucleotides difference) of the microsporidian isolate M1 to the sequence of *Microsporidium* sp. 515 ([GenBank: FN434086]) and 99.9% identity (1 nucleotide difference) of M2 to the sequence of *Microsporidium* sp. 505 ([GenBank: FN434085]), both isolated from Irish and French populations of *Gammarus duebeni celticus*[31]. The sequence derived from microsporidian M3 was 100% similar to a *Microsporidium* sp. defined by accession no. [GenBank: AJ438964] (*Microsporidium* sp. I), originally isolated from a UK population of *G. pulex*[14].

**Figure 1:**
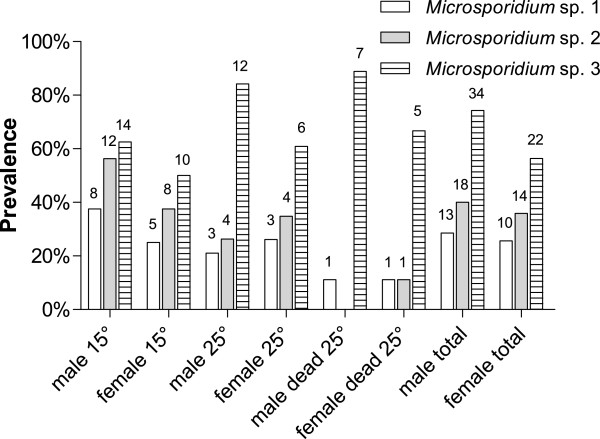
**Prevalences of *Microsporidium *spp. M1, M2 and M3 from *G. pulex *for males and females in all groups separately.** The female and male 25°C-groups include dead individuals. Numbers above bars indicate the corresponding number of individuals.

Prevalence of the microsporidians in gammarids recorded in the present study was high (M1: 27.0%, M2: 37.8% M3: 64.9%) and in total 82.4% of *G. pulex* were infected with one or all three microsporidians in a single host individual. Multiple infections (i.e. infections with all three microsporidian species in one individual gammarid) were detected in the *G. pulex* population with a prevalence of 8.1%. Bi-infections with M1 and M2 were found in 12.2% of all gammarids, with M1 and M3 in 17.6% and infections with M2 and M3 were detected with a prevalence of 25.7%. In general, the prevalence of M3 was higher in males (74.3%) compared to females (56.4%; see Figure 1), but no significant effect of infections on host sex was detected by GLM analysis for this or the other two species. Surprisingly, no M2-infected male was found among the individuals that died during the exposure to 25°C, and prevalence in females was markedly lower compared to the whole sample (females: 11.1% vs. 35.9%; males: 0% vs. 40.0%). In the same group, prevalence of M1 was equally low for both females and males (11.1% vs. 25.6% in females/28.6% in males). In contrast, M3 prevalence was higher in dead gammarids when compared to the total prevalence (females: 66.7% vs. 56.4%, males: 88.9% vs. 74.3%; see Figure 1). In the same group, three individuals (16.7%) were completely free of microsporidians.

### Hsp70 analysis

**Figure 2:**
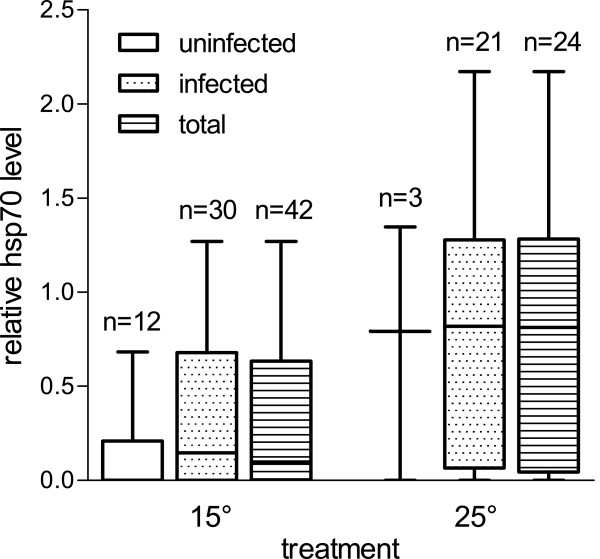
**Relative hsp70-levels in *G. pulex *kept at 15°C and 25°C (uninfected, infected and all individuals).** Boxes represent 25% percentiles, horizontal bar is the median and vertical bars give the range from minimum to maximum values. Effect of temperature on hsp70-level was significant according to analysis by LM (p = 0.0058, adjusted R^2^ = 0.43). “Infected” means microsporidian infection with *Microsporidium* sp. M1, M2 or M3 and any combination. Numbers above bars indicate the corresponding number of individuals.

**Figure 3:**
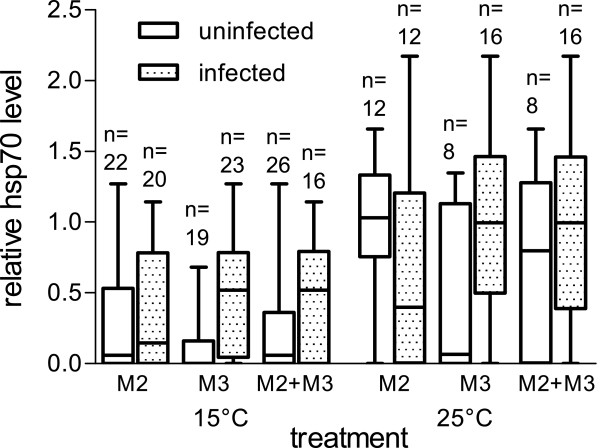
**Relative hsp70-levels in *G. pulex *individuals infected with *Microsporidium *sp. M2 and M3 kept at 15°C and 25°C.** Boxes represent 25% percentiles, horizontal bar is the median and vertical bars give the range from minimum to maximum values. M2 + M3 infected: individuals that are infected with both microsporidians M2 and M3 (can be infected with M1 as well); M2 + M3 uninfected: individuals that are not infected with microsporidian M2 and M3 together (can be uninfected, infected with M2 or M3 separately or M1 in alone or in combination with one of the other 2 species). Effect of infection with M2 + M3 on hsp70-level was significant in the 25°C group (p = 0.0470, adjusted R^2^ = 0.55) and in the whole sample (15°C and 25°C; adjusted R^2^ = 0.43; p = 0.0309) according to analysis by LM. Numbers above bars indicate the corresponding number of individuals.

Analysis by the linear model revealed a significant effect of temperature treatment (p = 0.0058, adjusted R^2^ = 0.43), as well as bi-infection with M2 + M3 (p = 0.0309) on hsp70 response. Figure 2 illustrates the significant effect of the treatment and the hsp70-response depending on infection status. When analyzing the 25°C data separately, the same interaction of M2 + M3 on hsp70 level was found (p = 0.0470, adjusted R^2^ = 0.55), together with a significant effect of gammarid wet weight (p = 0.0228). Figure 3 shows the hsp70 response in gammarids, depending on infection with microsporidians M2 and M3. No significant effects for any of the parameters were detected when analyzing the 15°C group separately.

**Figure 4:**
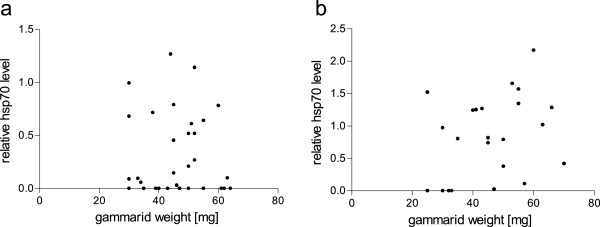
**Correlation of gammarid wet weight and relative hsp70 level. a)** 15°C group, **b)** 25°C group. Effect of gammarid weight was significant after 25°C exposure in analysis by LM (adjusted R^2^ = 0.55, p = 0.0228).

**Figure 5:**
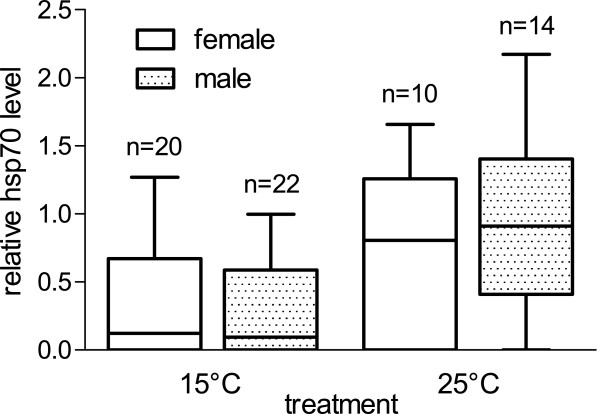
**Relative hsp70 level of *G. pulex *by sex and temperature.** Boxes represent 25% percentiles, horizontal bar is the median and vertical bars give the range from minimum to maximum values. Numbers above bars indicate the corresponding number of individuals.

Smaller gammarids seem to exhibit a weaker hsp70 response than larger ones when exposed to high temperatures, albeit this effect is not very strong (Figure 4). Host sex did not have a significant effect on the relative hsp70 level (Figure 5).

## Discussion

Temperature can significantly affect the dynamics of many parasite life cycles [35-37]. Also for microsporidians, the host-parasite interaction can be influenced by temperature. E.g. Kelley *et al.*[38] found that low temperatures impaired the feminizing effect of *Nosema granulosis* on their amphipod host and the transmission efficiency of the parasite. In the present study we report the effect of an experimental temperature increase on the stress response of *Gammarus pulex*, naturally infected with three microsporidian species.

To our knowledge, no morphological descriptions are available for any of the microsporidian species that were detected in *G. pulex* in the present study. Therefore, all three were placed in the catchall genus *Microsporidium* until they can be grouped taxonomically. The high similarity of the microsporidian partial ss rDNA sequences from the present study compared to isolates of Terry *et al.*[14] and Krebes *et al.*[31] indicates species identity, but this has to be substantiated by further analyses of more variable genetic markers as well as morphological and life cycle traits.

Previous molecular phylogenetic analyses suggested that M1 and M2 are closely related [31]. M3 was not used for the analysis in the latter study, but in the phylogeny of Terry *et al.*[14], M3 clustered in the same clade as M1/M2. This clade contains a mix of various genera (e.g. *Endoreticulatus*, *Glugoides*, *Vittaforma*, *Cryptosporogenes*) and is closely related to the genus *Nosema*. To our knowledge, the present study provides the first record of the microsporidians M1, M2 and M3 from Germany and shows their wide geographic distribution.

Some microsporidian species are highly host specific and seem to infect only a single gammarid species [39], whilst others are rather unspecific and can be found in different amphipod species [14]. The microsporidians M1 and M2, here detected in *G. pulex*, were both initially described from freshwater populations of *Gammarus duebeni celticus*[31] and are apparently not strictly host specific. Moreover, the samples for the study of Krebes *et al.*[31] were taken in Ireland, while a German population of gammarids was analyzed in the present study, indicating a wide geographic distribution of these parasites. *Microsporidium* sp. M3 was first described from a Scottish population of *G. pulex*[14] and might be more host specific, as it was not described from other gammarid species to date.

The prevalences of the microsporidians found in *G. pulex* in the present study ranged between 27%-64.9%. These prevalences are within the range reported for other microsporidians in natural populations of other amphipods, e.g. 46% (only females) for vertically transmitted *Nosema granulosis* in *G. duebeni*[40], 37%-85% prevalence of 2 microsporidians in females of *G. pseudolimnaeus*[41] or up to 27.1% in a Corophiid amphipod [13]. Infections with several microsporidian species in one individual amphipod are considered to be rare, and might only occur if horizontal transmission is present to some extent [42]. In our study, bi-and multiple infections were detected rather frequently with two or all three microsporidians. This indicates that horizontal transmission is the only or at least the predominant mode of transmission for M1 and M2. Contrary to these findings, Krebes *et al.*[31] did not detect bi-infections of *G. duebeni celticus* with M1 and M2 (*Microsporidium* sp. 515 and 505 in their study) what might be due to the use of universal primers for detection. The more abundant DNA in the sample will be amplified preferentially, and thereby infections of single host individuals with more than one parasite species remain undetected.

The male biased sex ratio observed in the gammarid population in the present study does not indicate the effect of a sex ratio distorting microsporidian, which would cause an excess of females in the population. For example, the sex ratio distorting microsporidian *Nosema granulosis* can reduce the median proportion of males in a *G. duebeni* population to less than 10% [11]. In *Microsporidium* sp. M3, vertical transmission was verified previously by screening of gammarid embryos for infection [14], but this parasite seems not to have a strong influence on the sex ratio of the host. The observed male biased population in the present study might be explained by the operational sex ratio that was previously found to be shifted towards males in gammarids [43,44].

Stress proteins like hsp70 are part of the repair mechanism that restores the functions of denatured proteins. Low hsp70 content in the cells would mean a decreased ability to repair denatured proteins under temperature stress, and could therefore impair survival. Surprisingly, small size of gammarids was not associated with higher mortality, as the mean weight of all gammarids was 47.5 mg and the mean weight of the dead individuals was 53.2 mg. The mortality at 25°C might not only be ascribed to the temperature increase, but might have been caused by decreasing oxygen concentrations in the water at higher temperatures.

The low prevalences of the microsporidians M1 and M2 in gammarids that died during exposure to high temperatures might suggest a protective effect of infection with these microsporidians against temperature stress. This finding seems to be in contrast to the high stress level indicated by hsp70 levels in individuals bi-infected with the microsporidians M2 and M3. One possible interpretation might be that microsporidian infections induced increased hsp70 levels that provided protection against a sudden stressor, like the temperature rise in the present study. Such direct or indirect positive fitness effects of parasitic infections for their hosts have been described in several cases [45]. Also for amphipods infected by microsporidians, positive effects for the host were reported. However, these effects were studied exclusively in vertically transmitted microsporidians, and included impairment of host fecundity [41], as well as reduction of number and survival of the offspring [11,13,42]. On the other hand the simultaneous occurrence of different stressors may also lead to additive stress effects causing exhaustion of the hsp70 response which might lead to death of these organisms.

In contrast to our findings, infection of *G. roeseli* with larvae of the acanthocephalan *Polymorphus minutus* reduced the hsp70 response in infected gammarids after palladium exposure or heat shock [24]*.* Moreover, in those gammarids that served as untreated controls, but were infected with the acanthocephalan larvae, no hsp70 could be detected at all. Recently, Frank *et al*. [25] showed that infection of *G. fossarum* with *P. minutus* resulted in higher hsp70 levels than in uninfected conspecifics, but following a 14 days cadmium exposure hsp70 levels in uninfected gammarids were four times higher than in infected *G. fossarum*. Both studies therefore indicate contrasting but significant effects of acanthocephalan larvae on hsp70 levels, at least if two stressors co-occur. With respect to microsporidians, Gismondi *et al*. [17] described a decrease in reduced glutathione, γ-glutamylcysteine ligase activity and metallothionein content in cadmium exposed and infected *G. roeseli* compared to gammarids which were not infected by the vertically transmitted microsporidians *Dictyocoela muelleri* and *D. roeselum*. Also, lipid and glycogen reserves were impaired by the combination of parasite infection and metal-exposure. Without cadmium, no effect of microsporidian infection on energy reserves and biomarker response was detected [17]. In the present study, we also found an increased response of the stress marker hsp70 to microsporidian infection. Interestingly, a significant parasite effect resulted from interaction of two microsporidian species, one a vertical transmitter, while the other is presumed to be horizontally transmitted. Usually, no severe pathogenic effects are expected for the host infected by vertically transmitted microsporidians [5]. Even under stressful conditions (food limitation and crowding) *G. duebeni* infected with the vertically transmitted microsporidian *N. granulosis,* did not suffer more compared to uninfected control individuals [15]. However, only cases of single infections were analyzed in these studies, while results of the present study show for the first time that multiple infections with different microsporidian species can have additive effects. Especially cases of bi-infections with vertically or horizontally transmitted microsporidians are of interest for the understanding of parasite population dynamics within a host population. Most horizontally transmitted microsporidians rely on high virulence, due to massive multiplication and spore formation causing host death, while vertically transmitted species depend on host survival, to allow reproduction and thereby transfer of the parasite to the next generation. The resulting conflict can be resolved by avoidance or sabotage of virulence [46], to allow both parasite species to coexist permanently in one host population. Sabotage of the host manipulation strategy of an acanthocephalan by a vertically transmitted microsporidian leading to decreased predation of the amphipod by the final host was reported by Haine *et al.*[46]. On the biochemical level, the results of Gismondi *et al.*[18] indicate a similar competitive effect of an acanthocephalan larvae and a vertically transmitted microsporidian, but only after cadmium exposure as additional stressor. Such a mutual influence between horizontally and vertically transmitted microsporidians (e.g. M1/M2 and M3 in the present study) might explain the coexistence of several microsporidian species in one host population.

## Conclusion

The present study provides first evidence for infection of a *Gammarus pulex* population from the River Ruhr with three species of microsporidians. Although the data has to be interpreted carefully due to uncontrolled factors in the naturally infected population (e.g. infection intensity and stage of parasite development) and the rather small sample size, the experimental data suggests that temperature and the combined effect of two of those microsporidians has an effect on the host stress level. Additionally, microsporidian infections might have a protective effect against temperature stress in some cases, by causing an elevated hsp70 level that is presumed to protect against temperature-mediated protein damage. However, if the added stress values reach a critical threshold the hsp70 response might be exhausted leading to more pathological effects than each stressor alone.

Further studies are planned to clarify the life cycle and transmission mode of the species M1 and M2 and to analyze the virulence of the microsporidians found in *G. pulex*. Additionally, comparisons of survival and fitness of single and multi-infected gammarids are required to elucidate the mechanisms, how the three microsporidians detected in the present study overcome the conflicts of horizontally and vertically transmitted parasites and manage to coexist in a single host population. These studies will also help to clarify the significance of possible protective effects of microsporidian infections against environmental stressors.

## Competing interests

The authors declare that they have no competing interests.

## Authors’ contributions

DSG conducted field sampling, species identification, temperature experiment, part of the laboratory work and data analysis. GS conducted most of the molecular biology (PCR and hsp70 analysis). BS and DSG had a substantial role in conception of the study, guidance of the practical work and writing the manuscript. All authors read and approved the manuscript.
